# Comparison of acute proton, photon, and low-dose priming effects on genes associated with extracellular matrix and adhesion molecules in the lungs

**DOI:** 10.1186/1755-1536-6-4

**Published:** 2013-02-04

**Authors:** Jian Tian, Sisi Tian, Daila S Gridley

**Affiliations:** 1 Department of Radiation Medicine, Radiation Research Laboratories and Department of Basic Sciences, Loma Linda University, Loma Linda, California, USA; 2 Department of Otolaryngology, Loma Linda University Medical Center, Loma Linda, California, 92354, USA; 3 Department of Pathological Anatomy, Nantong University, Nantong, China

## Abstract

**Background:**

Crew members on space missions inevitably are exposed to low background radiation and can receive much higher doses during solar particle events (SPE) that consist primarily of protons. Ionizing radiation could cause lung pathologies. Cell adhesion molecules (CAM) are believed to participate in fibrogenesis. Interactions between CAM and extracellular matrix (ECM) affect epithelial repair mechanisms in the lung. However, there are very limited data on biological effects of protons on normal lung tissue. Numerous reports have shown that exposure to low-dose/low-dose-rate (LDR) radiation can result in radioadaptation that renders cells more resistant to subsequent acute radiation. The goal of this study was to compare expression of genes associated with ECM and CAM, as well as critical profibrotic mediators, in mouse lungs after acute irradiation with photons and protons, and also determine whether pre-exposure to LDR γ-rays induces an adaptive effect.

**Results:**

Overall, a marked difference was present in the proton *vs.* photon groups in gene expression. When compared to 0 Gy, more genes were affected by protons than by photons at both time points (11 *vs.* 6 on day 21 and 14 *vs.* 8 on day 56), and all genes affected by protons were upregulated. Many genes were modulated by LDR γ-rays when combined with photons or protons. *Col1a1*, *mmp14*, and *mmp15* were significantly upregulated by all radiation regimens on day 21. Similarly, the change in expression of profibrotic proteins was also detected after acute and combination irradiation.

**Conclusion:**

These data show that marked differences were present between acutely delivered protons and photons in modulating genes, and the effect of protons was more profound than that of photons. Pre-exposure to LDR γ-rays ‘normalized’ some genes that were modified by acute irradiation.

## Background

Ionizing radiation (IR) includes photons, small packets of energy that carry electromagnetic radiation, as well as particle radiations such as protons. Among the forms of photon radiation, γ-rays have the smallest wavelength and the most energy of any other wave in the electromagnetic spectrum. In contrast, protons are subatomic particles with an electric charge of +1 and have greater biological impact compared to photons [1]. Virtually all forms of radiation are of concern to the National Aeronautics and Space Administration (NASA). Crew members on space missions are routinely exposed to low dose background radiation that is more than 150 times greater than on Earth [2] and are at risk for much higher doses during a solar particle event (SPE) that consists primarily of proton radiation [3]. Thus far, pulmonary abnormalities noted in astronauts have been attributed primarily to microgravity [4,5]; risk for lung complications associated with space-relevant radiation are unknown. There have been essentially no investigations that directly compare acute proton and photon effects on normal lung tissue and possible modification of the outcome due to low-dose/low-dose-rate (LDR) γ-ray pre-exposure. Better understanding of radiation effects on normal lung tissue also has relevance in the clinical setting. Unfortunately, patients who receive radiotherapy for lung cancer develop side effects such as inflammation and sometimes even fibrosis [6,7]. Protons, however, can be delivered to the tumor at a higher dose, while reducing the dose to normal tissue [8]. The physical advantage of a proton beam compared to conventional radiotherapy (X-rays) is that the beam can be modulated to deliver most of the dose to the intended target, that is, at the peak of the Bragg curve. With conventional radiation, the maximum dose is delivered within a few centimeters of the skin surface proximal to the target. Hence, proton radiation continues to be used with increasing frequency for the treatment of patients, including those with lung cancer [9].

The lungs are among the most radiosensitive organs in the body. Our previous investigations have shown that acute photon delivery resulted in profibrotic changes in the lungs of mice [10]. Lung repair is initiated immediately following injury and includes an acute inflammatory response, cytokine and growth factor release, activation of localized stem cells, and cell-cell and cell-matrix interactions mediated through cell adhesion molecules (CAM) [11]. Radiation-induced lung fibrosis (RILF), a major late effect of photon radiation damage [12], is characterized by loss of epithelia and excessive deposition of collagen and other extracellular matrix (ECM) components. CAM is believed to participate in fibrogenesis since relatively abundant CAM proteins and re-regulated mRNAs are detected in specimens of pulmonary fibrosis [13,14]. CAM-mediated adhesive interactions that may be involved in the pathogenesis include cell-ECM and cell-cell interactions that are mediated through several CAM families, including the integrins, cadherins, selectins, and members of the immunoglobulin superfamily. Therefore, stable adhesion during these interactions is essential for adequate cell communication, epithelial integrity, and ECM homeostasis.

Several evidences have shown that transforming growth factor (TGF)-β plays a primary role in the fibrotic process. During fibrogenesis, epithelial cells lose their characteristic markers such as E-cadherin responsible for their adhesion, and the expression of α-smooth muscle actin (SMA), a myofibroblast marker capable of producing abundant collagen and other ECM molecules [15,16], and Slug are enhanced. Slug (Snail 2) acts as a repressor of E-cadherin [17,18]. However, whether the expression of these markers for fibrogenesis are affected by protons or combination of irradiation with LDR-γ rays has not been identified to date.

Interestingly, an increasing number of studies have shown that exposure to low-dose radiation can result in radioadaptation that can be beneficial in that it renders cells more resistant to a subsequent acute radiation event, as well as more resistant to cancer and certain other pathologies [19,20]. The existence of this phenomenon, however, remains controversial. Thus, the overall focus of the present study was on lung parameters that may be altered due to excessive radiation exposure in the spaceflight environment, as well as nuclear/radiological events on Earth. Expression profiles of genes that are especially important in regeneration and remodeling of lung tissue after acute proton and photon irradiation were compared, both with and without pre-exposure to LDR γ-rays. In addition, protein profiles of a critical profibrotic cytokine and three markers for fibrogenesis were also compared between irradiated groups and the 0 Gray (Gy) control. The selection of day 21 post-irradiation rather than earlier time points was based in part on the premise that long-term changes in gene and protein expression patterns may have greater health-related significance than short-term changes that may simply be due to transient efforts to regain homeostasis. Day 56 was selected as the second time point to determine if the modulations observed in the lungs on day 21 were persistent. The present study is the first to investigate ECM and CAM response in the lungs under the conditions used.

## Results

### Histopathology

**Figure 1:**
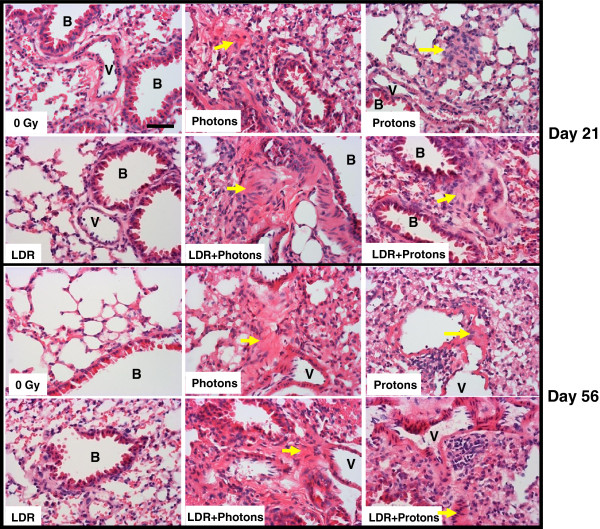
**Photomicrographs of representative histopathological profiles.** Sections of mouse lung tissue were paraffin-embedded post-irradiation and stained with hematoxylin and eosin in each cohort. Yellow arrows show fibrosis-like changes comprised of connective tissue and extracellular matrix present in samples from 0 Gy control and irradiated mice. B, bronchioles; V, vascular vessels. Magnification × 400, Bar = 20 μm.

**Figure 2:**
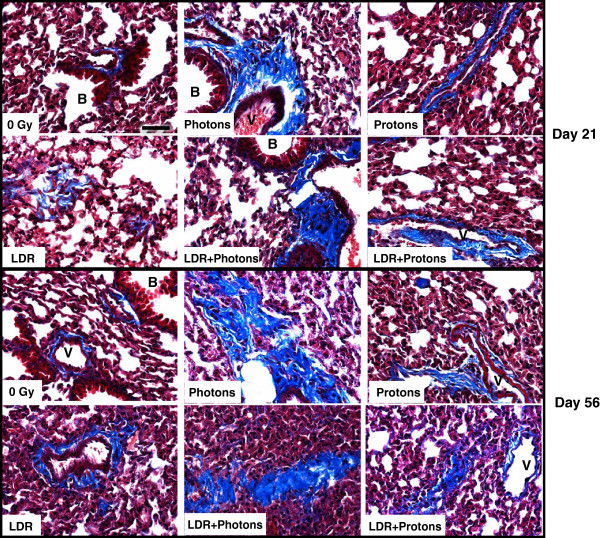
**Masson trichrome staining of lung sections exhibits collagen deposition (blue).** More abundant collagen is accumulated in alveolar space and at the sites surrounding vascular vessels or bronchioles in samples from Photon, Proton, LDR + Photon, and LDR + Proton groups as compared to the 0 Gy control and LDR samples. B, bronchioles; V, vascular vessels. Magnification × 400, Bar = 20 μm.

The histological appearance of lung tissue from control and LDR groups did not show marked abnormalities after staining with H&E. However, epithelial hyperplasia in bronchioles appeared to be present in some mice after acute exposure to 2 Gy photons or protons alone and in combination with LDR pre-exposure. The major adverse change was the presence of fibrosis-like lesions consisting of connective tissue components, ECM, and collagen in some acute and dual irradiation mice at both time points post-irradiation (Figure 1). Masson trichrome staining of lung sections to detect collagen deposition (blue color in Figure 2) showed more abundant collagen accumulated in alveolar spaces and at the sites surrounding vascular vessels or bronchioles in samples from Photon, Proton, LDR + Photon, and LDR + Proton groups as compared to the control and LDR samples.

### Gene expression profiles in irradiated groups compared to 0 Gy

mRNA levels of 84 relevant genes in the lungs were compared between photon and proton groups *vs*. the control on days 21 and 56. In addition, results from each of the groups receiving either photons or protons alone were also compared with their respective counterparts that were pre-exposed to LDR γ-rays. Table 1 presents the official symbol and brief description of the affected genes in the irradiated mice compared to 0 Gy (≥ 1.5-fold difference, *P* <0.05). The actual fold-change for these genes is shown in Table 2. We selected the 1.5-fold cutoff because there is no convincing evidence that a >2-fold difference always has a greater biological impact than a <2-fold difference; indeed, there is growing consensus that significant modulations that are <2-fold should be presented. Overall, more genes were affected by protons alone than by photons at both time points post-irradiation (11 *vs.* 6 on day 21 and 14 *vs.* 8 on day 56). Further analysis showed that all affected genes were up-regulated in the Proton group at both time points. Similarly, the majority of genes were also significantly upregulated by photons alone (Table 2).

**Table 1:** Symbols and description for all significantly modulated genes in irradiated groups compared to the 0 Gy control group

**Symbol**	**Name and gene category**
	*Cell adhesion molecules*
*cd44*	CD44 antigen
*cdh1*	Cadherin 1
*cdh3*	Cadherin 2
*ctgf*	Connective tissue growth factor
*fn1*	Fibronectin 1
*hapln1*	Hyaluronan & proreoglycan link protein 1
*itga2*	Integrin alpha 2
*itga3*	Integrin alpha 3
*itga4*	Integrin alpha 4
*Itgav*	Integrin alpha v
*Itgax*	Integrin alpha x
*itgb4*	Integriin beta 4
*ncam1*	Neural cell adhesion molecule 1
*ncam2*	Neural cell adhesion molecule 2
*pecam1*	platelet/endothelial cell adhesion molecule 1
*sele*	Selectin, endothelial cell
*sell*	Selectin, lymphocyte
*selp*	Selectin, platelet
*tgfbi*	Transforming growth factor, beta induced
	*Extracellular matrix proteins/proteinases*
*adamts1*	A disintegrin & metallopeptidase with thrombospondin type I motif 1
*adamts5*	A disintegrin & metallopeptidase with thrombospondin type I motif 5
*col1a1*	Collagen, type I, alpha 1
*col4a3*	Collagen, type IV, alpha 3
*col5a1*	Collagen, type V, alpha 1
*lama2*	Laminin, alpha 2
*lama3*	Laminin, alpha 3
*lamb3*	Laminin, beta 3
*lamc1*	Laminin, gamma 1
*mmp2*	Matrix metallopeptidase 2
*mmp7*	Matrix metallopeptidase 7
*mmp8*	Matrix metallopeptidase 8
*mmp11*	Matrix metallopeptidase 11
*mmp13*	Matrix metallopeptidase 13
*mmp14*	Matrix metallopeptidase 14
*mmp15*	Matrix metallopeptidase 15
*timp1*	Tissue inhibitor of metalloproteinase 1
*timp3*	Tissue inhibitor of metalloproteinase 3
*tnc*	Tenascin C

**Table 2:** **Genes with ≥1.5-fold difference and***P***<0.05 compared to 0 Gy on day 21 and 56 post-irradiation**

**Day 21**	**2 Gy photons**	**2 Gy protons**	**LDR**	**LDR + 2 Gy photons**	**LDR + 2 Gy protons**
*adamts5*	-	-	−1.58	-	-
*cdh3*	-	-	−2.49	-	-
*cd44*	-	1.94	-	1.88	1.67
*col1a1*	1.82	2.40	2.28	2.52	1.58
*col4a3*	-	-	-	1.69	1.61
*col5a1*	-	-	-	1.81	1.60
*fn1*	-	-	-	1.53	-
*hapln1*	-	1.96	-	-	1.87
*itga2*	-	1.51	-	1.57	-
*itga3*	-	-	-	1.61	-
*Itgav*	-	1.63	-	1.53	1.57
*Itgax*	-	-	-	1.53	-
*lama2*	1.71	-	-	-	-
*lama3*	-	-	-	1.77	-
*lamb3*	-	1.62	-	-	1.79
*mmp2*	-	-	−1.62	-	-
*mmp7*	−2.46	-	-	-	-
*mmp11*	-	-		-	1.87
*mmp14*	1.61	1.63	1.62	1.94	1.59
*mmp15*	1.50	1.66	1.61	1.96	1.78
*ncam2*	-	-	-	2.11	-
*selp*	-	-	-	1.56	1.56
*tgfbi*	-	-	-	-	−1.50
*timp1*	1.59	1.89	-	-	-
*timp3*	-	2.44	-	1.98	-
*tnc*	-	1.75	-	1.61	-
Total (26)	6	11	6	16	12
**Day 56**	**2 Gy photons**	**2 Gy protons**	**LDR**	**LDR + 2 Gy photons**	**LDR + 2 Gy protons**
*adamts1*	-	-	-	1.57	-
*cdh1*	-	-	-	1.71	-
*cdh3*	2.97	4.02	-	3.98	-
*cd44*	-	1.66	-	-	-
*col1a1*	-	1.67	-	-	2.31
*col4a3*	1.64	-	-	1.69	-
*ctgf*	-	-	-	1.70	-
*itga2*	1.54	1.72	-	-	-
*itga4*	−1.63	-	−1.82	−1.52	−2.09
*itgax*	-	1.55	-	-	-
*itgb4*	-	1.86	1.74	-	1.71
*lama2*	-	-	-	1.74	-
*lamb3*	-	1.58	-	-	-
*lamc1*	-	1.60	-	-	-
*mmp8*	-	-	-	1.76	-
*mmp13*	-	-	-	-	−2.21
*mmp14*	1.55	1.75	-	-	1.66
*mmp15*	1.54	1.79	-	-	1.56
*ncam1*	-	1.94	-	-	-
*ncam2*	2.41	-	-	3.6	-
*pecam1*	-	-	-	3.46	-
*sele*	-	-	1.57	-	-
*sell*	-	-	-	-	−1.94
*tgfbi*	-	1.56	-	-	-
*timp3*	3.70	4.00	-	4.10	-
*tnc*	-	1.56	-	-	-
Total (26)	8	14	3	11	7

Gene expression in lung samples was determined using quantitative RT-PCT. The numbers represents the fold-changes based on each irradiated group *versus* control group.

-, *P* value was >0.05 and/or relative fold-change was <1.5.

Furthermore, the data showed that pre-exposure to LDR γ-rays increased the number of affected genes compared to acute photons alone (16 *vs*. 6 on day 21 and 11 *vs.* 8 on day 56). In the LDR + Photon group, 100% and about 91% of the affected genes were upregulated on days 21 and 56, respectively. Unlike the effect of LDR pre-exposure on response to subsequent 2 Gy photons, only one more gene was affected in the LDR + Proton group on day 21 as compared to protons alone (12 *vs.* 11). Surprisingly, only 50% of the genes that were modified by protons alone were affected in the LDR + Proton group on day 56 (7 *vs*. 14). More importantly, LDR pre-exposure ‘normalized’ the expression of several genes that were up-/downregulated by acute delivery of either protons or photons (that is, the levels of up- or downregulated genes by acute radiation were similar to those of 0 Gy control due to LDR pre-exposure). At both time points of assessment, expression of three genes in the Photon group were equivalent to 0 Gy due to LDR pre-exposure. In the Proton group, four and 10 genes were ‘normalized’ on days 21 (4/11) and 56 (10/14). On the other hand, some genes that were up-/downregulated in the combination groups were equivalent to 0 Gy when acute photons or protons were delivered alone (Table 2).

Further analyses showed that eight common genes were significantly upregulated in both combination irradiation groups (8/16 in LDR + Photon and 8/12 in LDR + Proton) as compared with 0 Gy on day 21 (Table 2). In contrast, only *itga4* was downregulated in both combination irradiation groups on day 56. When comparisons were performed between acute proton or photon delivery alone with their respective counterparts that were pre-exposed to LDR γ-rays, only three genes were affected by photons alone (3/6) *versus* LDR plus photons (3/16), and seven genes were upregulated by protons alone (7/11) *versus* LDR plus protons (7/12) on day 21. On day 56, five genes were modulated by acute photons alone as well as in combination with LDR; in the Proton and LDR + Proton groups, the same four genes were affected.

Among 26 genes that were modulated on day 21, only *col1a1*, *mmp14*, and *mmp15* were significantly upregulated by all radiation regimens. On day 56, however, *col1a1* was upregulated only by proton radiation; *mmp14 and mmp15* were ‘normalized’ in the LDR and LDR + Photon groups. Interestingly, in all four groups that received γ-ray photons (LDR, Photons, LDR + Photons, and LDR + Protons), the *itga4* gene was significantly downregulated on day 56.

### Comparison of gene expression profiles among irradiated groups

When comparison was made between 2 Gy protons *versus* 2 Gy photons, expression of six genes was significantly different in the Proton group on day 21 (five upregulated and one downregulated) and only one was upregulated on day 56 (Table 3). In addition, on day 21 there were three genes in the LDR + Photon group that were upregulated compared to the Photon group, whereas only one gene was upregulated in the LDR + Proton group *versus* protons alone (no genes were downregulated). By day 56, expression of three genes in the LDR + Photon group was different compared to the Photon group (two upregulated and one downregulated). In the LDR + Proton group at this same time point, expression of only one gene (downregulated) was different from proton radiation alone. We also include all genes that were not affected post-irradiation (Table 4).

**Table 3:** **Genes with ≥1.5-fold and***P***<0.05 between irradiated groups on days 21 and 56 post-irradiation**

**Day 21**	**Protons *vs. *Photons**	**LDR + Photons *vs. *Photons**	**LDR + Protons *vs. *Protons**	**LDR + Protons *vs. *LDR + Photons**
*col1a1*	-	-	−1.52	−1.6
*itgb4*	−1.54	-	-	-
*lama2*	1.86	1.56	-	-
*mmp1a*	1.50	-	-	1.55
*ncam2*	2.04	2.91	-	-
*timp3*	2.17	1.75	−2.33	-
*vcam1*	1.94	-	-	-
Total (7)	6	3	2	2
**Day 56**	**Protons *vs. *Photons**	**LDR + Photons *vs. *Photons**	**LDR + Protons *vs. *Protons**	**LDR + Protons *vs. *LDR + Photons**
*adamts1*	-	1.50	-	-
*col1a1*	-	−1.51	-	2.52
*ctgf*	-	1.5	-	-
*mmp9*	-	-	-	−1.64
*ncam2*	-	-	-	−2.36
*sell*	-	-	−1.97	-
*sgce*	-	-	-	−1.68
*spock1*	2.09	-	-	-
Total (8)	1	3	1	4

Gene expression in lung samples was determined using quantitative RT-PCT. The numbers represents the fold-changes based on each irradiated group *versus* control group.

-,*P* value was >0.05 and/or relative fold-change was <1.5.

**Table 4:** Among 84 analyzed genes that were not affected by radiation exposure

**Symbol**	**Name**
*adamts2*	A disintegrin-like and metallopeptidease with thrombospondin type 1 motif, 2
*adamts8*	A disintegrin-like and metallopeptidease with thrombospondin type 1 motif, 8
*cdh1*	cadherin 1
*cdh4*	cadherin 4
*ctnna1*	catenin (cadherin associated protein), alpha 1
*ctnna2*	catenin (cadherin associated protein), alpha 2
*ctnnb1*	catenin (cadherin associated protein), beta 1
*cntn 1*	contactin 1
*col2a1*	collagen, Type II, alpha 1
*col3a1*	collagen, Type III, alpha 1
*col4a1*	collagen, Type IV, alpha 1
*col4a2*	collagen, Type IV, alpha 2
*col6a1*	collagen, Type VI, alpha 1
*ecm1*	extracellular matrix protein 1
*emifin1*	elastin microfibril interfacer 1
*entpd1*	Ectonucleoside triphosphate diphosphohydrolase 1
*fbln1*	fibulin 1
*hapln1*	hyaluronan and proteoglycan link protein 1
*Hc*	hemolytic complement
*icam1*	intercellular adhesion molecule 1
*itga5*	integrin alpha 5
*Itgae*	integrin alpha e
*Itgal*	integrin alpha l
*Itgam*	integrin alpha m
*itgb1*	integrin beta 1
*itgb2*	integrin beta 2
*itgb3*	integrin beta 3
*itgb4*	integrin beta 4
*lama1*	laminin, alpha 1
*lamb2*	laminin, beta 2
*lamb3*	laminin, beta 3
*mmp3*	matrix metallopeptidase 3
*postn*	periostin, osteoblast specific factor
*sparc*	secreted acidic cysteine rich glycoprotein
*spp1*	secreted phosphoprotein 1
*syt1*	synaptotagmin 1
*thbs1*	thrombospondin 1
*thbs2*	thrombospondin 2
*thbs3*	thrombospondin 3
*vcan*	Versican
*vtn*	Vitronectin

### Expression of profibrotic proteins

Because the panel of 84 genes related to ECM and CAM did not include some other critical profibrotic factors, we also evaluated expression profiles of four major proteins: a cytokine frequently associated with fibrogenesis (TGF-β1) and three other markers for mesenchymal cells or that are known to be involved in EMT.

**Figure 3:**
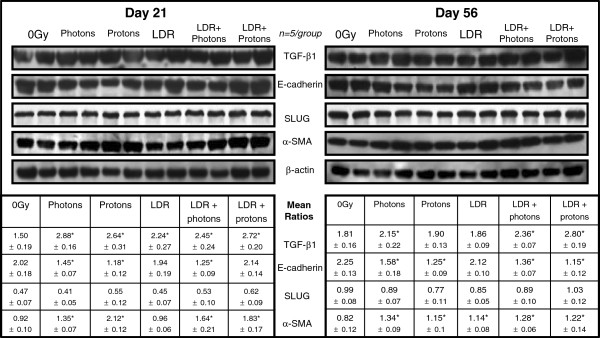
**Representative blots of four profibrotic mediators in lung tissue.** Expression of the proteins (five samples/group) and β-actin was determined by Western blotting. The tables show the ratios of target bands to β-actin corresponding to western blots and analyzed with two-way ANOVA. *P <0.05 compared to 0 Gy at the corresponding time point; means +/− standard error of the means (SEM) are shown. LDR, low-dose/low-dose-rate.

TGF-β1 is a critical cytokine. Western blotting showed that its expression was significantly enhanced in response to all radiation regimens at day 21 and to photons and both dual irradiations on day 56 as compared to the non-irradiated group (*P* <0.05) (Figure 3).

E-cadherin expressed in epithelial cells is responsible for cell-cell adhesion. Statistical analysis of Western blotting showed that the mean ratios of this adhesion molecule to β-actin were significantly reduced in samples from the two acutely irradiated and LDR + Photon groups at both time points and in the LDR + Proton group on day 56 post-irradiation (*P* <0.05) (Figure 3).

Slug is a marker for EMT. It is involved in E-cadherin repression and also has antiapoptotic activity. Western blotting did not reveal marked enhancement or decrease in expression of this protein between groups.

α-SMA is expressed only in mesenchymal cells that produce collagen and ECM. Western blotting showed that α-SMA was significantly increased in all irradiated mice at both time points except on day 21 in the group that received LDR radiation alone (Figure 3).

## Discussion

The lung is dynamically remodeled in response to injury, in which the change in ECM compositions can lead to either healthy or impaired regeneration. The tissue integrity and cell-cell or cell-matrix communications rely on cell adhesion molecules. Since protons and photons are two different forms of radiation, the biological effects generated by them are likely to be different. In this study, the changes in histology indicated that both types of radiations can induce profibrotic responses. Increased deposition of collagen in the lungs from irradiated animals (Figures 1 and 2) implies that activation of collagen producing cells is accelerated, leading to fibrosis-like change. Many studies have shown that low-dose radiation can induce radioadaptation that renders cells more resistant to a subsequent acute radiation event [20]. In this study, accumulation of more abundant collagen in both groups that received dual irradiation indicates that pre-exposure to LDR γ-rays did not render normal lung tissue more resistant to excessive production of this protein caused by acute radiation exposure.

In the present study, the differences between protons and photons in modulating expression of the genes related to ECM or CAM indicated that sensitivity of these genes to the two types of acute radiation regimens was different. More genes were affected by protons than by photons at both time points, suggesting that these relevant genes are more susceptible to protons. The markedly higher number of genes that were upregulated in the LDR + Photon group at both time points compared to the one that received only acute photons (Table 2) indicates that the protracted low-dose priming with γ-rays triggered mechanisms that rendered genes more responsive to acute photons. Only three genes (*col1a1*, *mmp-14*, and *mmp-15*) were upregulated in all irradiated groups on day 21, indicating that sensitivity of most genes was dependent on the type of radiation regimen. *Col1a1* encodes one of the markers for mesenchymal cell lineage. In addition, overexpression of matrix metalloproteinase (MMP) family members such as MMP-14 and MMP-15 is a consequence of perturbation of the balance between synthesis and degradation of collagen and other ECM components [21]. MMP-14 is capable of proteolytic degradation of type I, II, and III collagens following the characteristic cleavage pathway [22]. MMP-14 also cleaves many membrane-anchored proteins such as E- and N-cadherin, integrins, CD44 (a hyaluronan receptor), and several cell surface proteoglycans and their receptors [22]. MMP-15 is a ubiquitously expressed enzyme with largely overlapping substrate specificity with MMP-14 [23]. The profibrotic change in histopathology post-irradiation indicates that all radiation regimens used in the present study resulted in perturbation of normal tissue remodeling and excessive production of collagen. The overproduction of collagen and/or ECM may initiate transcription of genes that downregulate MMP, that is, MMP inhibitor genes, timp-1 and/or timp-3. Of course, the final fate of lung status after irradiation depends on whether normal or aberrant repair takes place, a process that is largely determined by cell microenvironment.

In addition, *cd44* and *itgav* (encoding a αv subunit of integrin) were significantly upregulated in the Proton and both combination groups on day 21. CD44 mediates cell-cell and cell-matrix interactions through its affinity for hyaluronic acid (HA), and also other ligands such as collagens and MMPs. Therefore, altered expression or dysfunction of CD44 may cause pathogenic phenotypes. The *itgav* gene encodes a receptor for many ligands, such as fibronectin, fibrinogen, laminin, and MMP-2. Moreover, the αv subunit can also activate TGF-β1 when it is associated with a β6 or β8 subunit on airway epithelial cells, leading to poor wound healing [24]. When cell surface proteins like E-cadherin and integrins that mediate epithelial connections to neighboring cells and the basement membrane, respectively, are replaced by N-cadherin and ectopic integrins, the cell may be primed for the mesenchymal phenotype by more transient adhesive properties [25]. In addition, we also noted that the two combined radiation groups had more upregulated integrin genes that participate in cell-matrix adhesion and/or are collagen and ECM structure constituents. This was especially evident on day 21 and suggests that the combined irradiation may also cause dysregulated repair. In addition, significant downregulation of *itga4* by γ-rays, either at a high dose or a low dose, indicates that this gene was highly sensitive to γ-ray photons.

Two collagen genes (*col4a3* and *col5a1*) and *selp* (P-selectin, platelet) that were significantly upregulated on day 21 after protracted exposure to LDR γ-rays combined with acute radiation further indicates that synthesis of collagen may be enhanced. P-selectin is an inflammatory adhesion molecule found on endothelial cells and platelets. It enables the recruitment of leukocytes to the endothelium and activates platelets. Platelets contain a plethora of growth factors and cytokines, including high concentrations of TGF-β [26] that is well known to be a strong inducer of fibrogenesis. Thus, upregulation of *selp* can lead to the release of more cytokines and growth factors from activated platelets, potentially promoting a mesenchymal phenotype [27].

The enhanced appearance of major profibrotic proteins in response to the radiation regimens also indicates epithelial early injury. Here we noted excess production of collagen and ECM molecules after acute and combination irradiation. It is well established that TGF-β1 promotes differentiation of fibroblasts into activated myofibroblasts, enhances collagen synthesis, and reduces collagen degradation by downregulating proteases and upregulating protease inhibitors [28]. Increased expression of TGF-β1 in the samples from irradiated groups at both time points indicates that the mechanisms responsible for production of this cytokine are very sensitive to ionizing radiation, even low-dose-rate photons delivered over an extended time period.

Myofibroblasts are strongly associated with tissue repair and fibrosis. α-SMA, a major marker of myofibroblasts, is linked to cell-cell adherence sites and cell-matrix anchorages, the latter being essential for contractile as well as biosynthetic functions. The changes in histology and collagen staining in the irradiated groups may partly result from transition of more myofibroblasts from other cell types, for example, epithelial or adult stem cells. TGF-β1 is one of several profibrotic cytokines that activate myofibroblast progenitors [29]. As a result, excessive accumulation of collagen and ECM can occur. Reactive oxygen species (ROS) generated by radiation exposure are immediate activators of TGF-β1 [30,31]. Evidence suggests that once activated, myofibroblasts may not require continued paracrine stimulation by fibrogenic cytokines, relying instead on positive autocrine feedback [29].

E-cadherin is broadly expressed only by epithelial cells. It is also a key component of adhesion junctions that play an important role in maintenance of epithelial integrity [32]. In this study, low ratio of E-cadherin to β-actin bands indicates that this epithelial marker was sensitive to radiation. Low E-cadherin is associated with breakdown of epithelial integrity and also poor repair capacity.

Jayachandran *et al*. [33] showed that TGF-β1 treatment increased the expression and nuclear accumulation of Slug, a transcription factor, in concert with induction of EMT in type II alveolar cells. However, in the present study, Slug expression was not markedly affected by ionizing radiation at the two time points of assessment, thereby implying that Slug protein, unlike TGF-β1, E-cadherin, and α-SMA, was not sensitive to irradiation. Further study will explore the expression of Slug gene post-irradiation.

## Conclusion

This study provides new information on normal lung response to proton radiation. Both acute proton and photon radiation regimens induced overexpression of certain ECM/CAM genes and profibrotic mediators, leading to production of excess collagen and ECM. The present data also showed that the effect of protons on gene expression was more profound compared to photons. The differences in gene expression that were noted between the acutely irradiated groups are likely due to the different physical properties of protons and photons. Protons are particles with a positive electrical charge equal to that of electrons, whereas photons are a type of electromagnetic radiation consisting of elementary particles. Furthermore, pre-exposure to LDR γ-rays resulted in more modification in the gene profile when followed by acute photon irradiation compared to acute protons. These findings have implications related to human space exploration, as well as radiation exposure above normal on Earth. Future studies should include more time points and multiple doses after exposure to both forms of ionizing radiation. Furthermore, since fibrosis is an unlikely outcome after a 2 Gy dose as used here, future studies should include higher radiation doses localized to the lungs and longer intervals post-irradiation to determine if a difference exists between photons and protons in ultimate outcome and if LDR pre-exposure has modifying effects.

## Methods

### Animals and study design

Wild-type C57BL/6 mice (*n* = 120, 8 to 9-week-old female mice; Charles River Laboratories, Hollister, CA, USA) were maintained under standard vivarium conditions in a BioZone VentiRack™ (BioZone, Inc., Fort Mill, SC, USA). There were six groups: (1) 0 Gy; (2) Photons; (3) Protons; (4) LDR; (5) LDR + Photons; and (6) LDR + Protons. Subsets/groups were euthanized in 100% CO_2_ on days 21 and 56 post-irradiation. The protocol was approved by the Loma Linda University (LLU) Institutional Animal Care and Use Committee prior to initiation and followed all recommendations in the Guide for the Care and Use of Laboratory Animals of the National Institutes of Health. The mice were monitored daily by expert LLU animal care personnel and the investigators. All efforts were made to ensure little or no suffering.

### Irradiation procedures

For LDR priming, mice were irradiated using ^57^Co plates (AEA Technology, Burlington, MA, USA) placed immediately beneath their cages (one plate/two cages in BioZone rack). A total dose of 0.01 Gy was delivered at 0.03 cGy/h to a maximum of five mice/large cage. Since γ-rays easily penetrate for long distances through many materials (major exceptions being lead and concrete), the dose for each individual animal was not determined. Dose confirmation, however, was performed using several thermoluminescent dosimeters (TLD) at various locations per cage. The data showed that dose uniformity was ± 5%.

For acute photon and proton irradiation, mice were placed individually into polystyrene aerated cubicles and irradiated to 2 Gy once within a very short period of time. Photon radiation was delivered using ^60^Co γ-rays (0.8 Gy/min; Eldorado Model G, Atomic Energy of Canada Ltd, Ottawa, Canada). Proton irradiation was done in the Proton Treatment and Research Center at Loma Linda University Medical Center; an average dose rate of 0.9 Gy/min and 230 MeV energy was used. Different groups received acute photon or proton radiation alone or within 1–2 h after protracted exposure to LDR γ-rays. Additional details on set-up and dosimetry have been previously published [20].

### Analysis of gene expression by quantitative reverse transcriptase-polymerase chain reaction (RT-PCR)

Immediately after sacrifice, the right lungs were frozen in liquid nitrogen (*n* = 5 mice/group/time point) and kept at −80°C until analysis. The 84 relevant genes were assessed using RT^2^ Profiler™ PCR Array PAMM-013 (SuperArray BioSciences, Frederick, MD, USA) to compare mRNA levels of ECM and CAM in lung tissue between irradiated groups and the 0 Gy control. To determine the effect of LDR γ-rays on response to acute radiation, gene expression levels in the LDR + Photon and LDR + Proton groups were compared to the levels obtained for their counterparts that received acute radiation alone. RNA extraction, RT-PCR, and its data analyses were performed by SuperArray BioSciences. Genes with ≥1.5-fold difference in expression and *P* <0.05 in the various group comparisons are emphasized. The ΔΔCt method used in PCR array of this study is widely accepted and known as a valid method for relative quantification of real-time PCR data.

### Histopathology

After harvest, the left lungs were formalin-fixed, paraffin-embedded, and sections (5-μm thick) were prepared for standard hematoxylin/eosin (H&E) and Masson trichrome collagen staining. The changes in histology were assessed under a light microscope.

### Masson Trichrome collagen staining

Briefly, deparaffinzed sections were pretreated in Bouin’s fluid at 56°C for 1 h before they were stained with Weigert’s Iron Hematoxylin, Biebrich Scarlet-Acid Fuchsin solution and Aniline Blue Stain Solution. Finally, 1% Acetic Acid solution was used to remove non-specific staining. After this procedure, the nuclei are stained black, cytoplasm and muscle fibers are displayed as red, and blue color represents collagen. The lung consists of a great number of alveoli and its expansion is not uniform throughout. Although, in this study, whole-mount sections of the lungs were not scanned for quantification of collagen, we could identify the difference in relative amounts of collagen in lung tissue from the Trichrome stained sections under a microscope.

### Western blotting

A total of 50 mg of lung tissue from each mouse was homogenized in 50 μL of RIPA lysis buffer (Sigma, St. Louis, MO, USA) containing protease inhibitors (Roche, Indianapolis, IN, USA), and then pelleted via centrifugation at 13,000 g at 4°C. The protein concentration in supernatants was measured using the Coomassie Plus assay (Pierce, Rockford, IL, USA). Then, 25 mg of protein was loaded in each well of the gel for electrophoresis prior to electroblotting proteins onto polyvinylidene fluoride (PVDF) membranes (Invitrogen, Carlsbad, CA, USA). In order to compare marker expression under the same condition, we loaded two of five samples from each group on the same gel with 12 wells. Six gels were used for a total of 60 specimens (five mice/group/time point). All membranes were incubated overnight with primary antibody at 4°C following blocking non-specific binding sites in 5% non-fat milk in Tris Buffered Saline (TBS) containing 0.1% Tween 20. The same membranes after electroblotting were incubated separately with different primary antibodies that were used to detect the proteins with different size prior to treatment with the secondary antibody conjugated with horseradish peroxidase (HRP, Millipore, Temecula, CA, USA). However, when detecting different proteins with similar size, stripping buffer (2% SDS, 62.5 mM Tris–HCl pH 6.7, 100 mM 2-mercaptoethanol) was used to remove primary and secondary antibodies on the PVDF membrane before proceeding to detect another target. This stripping buffer removes antibodies from membranes very effectively. Nonetheless, every time the stripped membranes were incubated with second antibody to confirm that no previous primary antibody remained on the membranes prior to incubating them with a new primary antibody. Four rabbit anti-mouse primary polyclonal antibodies were used to identify transforming growth factor (TGF)-β1 (recombinant full length inactive form), E-cadherin, Slug, and α-SMA. β-actin, utilized as a housekeeping protein, was detected with polyclonal antibody. All antibodies were purchased from Abcam. The enhanced chemiluminescence (ECL) and film exposure method (GE Healthcare-Bio-Sciences Corp., Piscataway, NJ, USA) was used to display the bands on the membranes. We used the Biospectrum 310 MultiSpectral System (Ultra-Violet Products, Ltd., Upland, CA, USA) and the appropriate software to measure band intensity on the films. This device is able to select real size of individual target bands, thus eliminating as much background as possible.

### Statistical analysis

Gene expression data were evaluated using Student’s t-test, a well-accepted method for data obtained using the RT-PCR method described above. The ratios of target bands to β-actin obtained from Western blotting between each of the irradiated groups and 0 Gy at both time points was analyzed using two-way analysis of variance (ANOVA) followed by the Tukey test (Systat Software, Richmond, CA, USA).

## Abbreviations

α-SMA: Alpha-smooth muscle actin; CAM: Cell adhesion molecules; ECM: Extracellular matrix; EMT: Epithelial-mesenchymal transition; Gy: Gray; IR: Ionizing radiation; LDR: Low-dose/Low-dose-rate; RILF: Radiation-induced lung fibrosis; RT-PCR: Reverse transcriptase-polymerase chain reaction; TGF-β1: Transforming growth factor-beta1.

## Competing interests

No competing interests are declared by all authors.

## Authors’ contributions

JT and ST carried out the experiments and performed the statistical analyses reported in this study. JT and DSG conceived the study, participated in the design of the study, participated in its coordination, and drafted the manuscript. All authors read and approved the final manuscript.
